# Paediatric emergency department visits for non-urgent conditions: Can family medicine prevent this?

**DOI:** 10.1080/13814788.2020.1825676

**Published:** 2020-10-07

**Authors:** Hatice Tuba Akbayram, Enes Coskun

**Affiliations:** a Department of Family Medicine, Gaziantep University Faculty of Medicine, Gaziantep, Turkey; b Department of Pediatrics, Gaziantep University Faculty of Medicine, Gaziantep, Turkey

**Keywords:** Paediatric emergency department, non-urgent, family physician, primary care

## Abstract

**Background:**

In Turkey, family physicians serve only during office hours, while emergency services have 7/24 free access. Non-urgent patients commonly use Paediatric Emergency departments (PEDs). In Turkey, there is little evidence as to why emergency services are used instead of family medicine for non-urgent paediatric healthcare.

**Objectives:**

To evaluate the causes and factors affecting non-urgent PED visits. To determine the reason for non-use of family medicine for non-urgent paediatric healthcare.

**Methods:**

We conducted a cross-sectional study at Gaziantep University PED between April and May 2019. We administered a questionnaire to the parents of children (from one month to 16 years) triaged to non-urgent (level-5) using a 5-level triage system.

**Results:**

A total of 457 parents were surveyed. The average patient age was 6.5 ± 4.7 years and 24.5% had a chronic disease. One-third of the parents (33.7%) considered their children’s condition ‘very urgent’. The most important reason for preferring PED (42.5%) instead of family physician or alternative health facilities was the thought that the condition of children would worsen. Two hundred fifty-three (55.4%) of the patients presented outside working hours. Although 58.9% of parents were satisfied with the family physician, most (67.8%) stated that they preferred other specialists rather than family physicians when the child had health problems. Fathers who were primary school graduates were more likely to prefer other specialists than family physicians.

**Conclusion:**

Parents’ perception of urgency and the thought that their child’s condition will worsen are the main reasons for non-urgent using PED.

## Introduction

KEY MESSAGES

- Pediatric emergency departments due to visits by non-urgent patients is an important problem.
- The number of non-urgent visits to pediatric emergency departments can be reduced by training and informing parents on pediatric health and diseases, using family physicians as the point of first contact.

Overcrowded emergency departments (EDs) and paediatric emergency departments (PEDs) due to visits by non-urgent patients is an important problem in Turkey and throughout the world [1,2]. Taking children to ED, even though they have a health problem that can be handled by family physicians or primary healthcare centres, leads to overcrowded PED [3]. Non-urgent paediatric patients are more frequently brought to PED on the weekends and at night, when primary healthcare centres are closed [4]. Overcrowding in ED causes increased waiting times, delayed treatment services, decreased patient satisfaction, financial burden, and increased morbidity and mortality [5].

Non-urgent patient visits are typically defined as visits for conditions for which a delay of several hours would not increase the likelihood of an adverse outcome [6]. The rate of non-urgent patients presenting to EDs ranges between 40 and 58% according to previous studies [7–9]. According to the 2017 data of the Turkish Ministry of Health, the number of visits to PED was nearly 7.7 million within the first nine months of 2017, and these visits constituted 2.61% of all visits to outpatient clinics [10]. However, it has not been reported how much of these visits are made up of non-urgent patients.

There may be many factors contributing to why patients with non-urgent health problems visit the ED before their primary care physician. These factors include child’s age, parents’ thoughts about their child’s need for urgent care and worsening of the child’s health, perceived advantages of ED, perception of other healthcare services, lack of primary care access and low health literacy [3,4,11]. Moreover, the perception that primary healthcare services are worse than the services provided in the ED and dissatisfaction with the diagnosis and treatment provided by primary care physicians are important factors [12,13].

In Turkey, primary healthcare services have been provided by family physicians since 2010 [14]. Only 8% of family physicians are family medicine specialists; the vast majority are certified general practitioners [15]. A family physician is in general, available during office hours (8 a.m. to 5 p.m., workdays). Primary healthcare is provided at EDs during non-working hours. Turkey has not yet established a referral system. Parents can take their children directly to healthcare facilities and emergency services. The mean number of yearly contacts with the primary healthcare facilities in Turkey is 3.2 per person. However, the number of applications for secondary and tertiary health institutions is 6.3 [15].

ED triage allows the opportunity to identify high-risk patients rapidly. Regarding the triage system used in Turkey, the patients are classified as red (level 1 and 2), yellow (level 3 and 4) and green (level 5). Level 5 patients are in the lowest risk group and do not need urgent intervention [16].

Parents are the determinant of the choice of medical assistance for their children. To develop solutions for the over crowdedness of ED, it is important to evaluate the reasons behind the parent’s preference of ED for the treatment of their children instead of the family physician. Studies on this subject in Turkey are scarce. In this study, we aimed to determine the reasons for PED visit, the factors affecting this and to find out the rationale behind parents’ attitude of not preferring family medicine for non-urgent paediatric care.

## Methods

### Study design and setting

This cross-sectional study was conducted between April 2019 and May 2019 in the PED of Gaziantep University Medical Faculty. This hospital is one of the leading tertiary healthcare institutions in south Turkey. The PED of the hospital provides services to nearly 54,000 patients each year.

The five-level triage system was in use at the PED in which the study was conducted. Trained nurses are in charge of classifying children to different priority groups. The non-urgent patient (level-5) is a stable patient with simple symptoms or signs that do not require further intervention.

Parents of children whose age is between 1 month and 16 years old and who were assigned level 5 of the triage category by a triage nurse participated in the study. Parents of children who do not speak Turkish and/or who have any diagnosis of trauma, poisoning and/or forensic situation and/or who were assigned level 1–2–3–4 of triage category by a nurse were excluded from the study.

### Survey form and data collection

The questionnaire, contained 19 questions, divided into 2 sections. The first section was on demographic characteristics of the family (parent’s age, parent’s education status, income status and child’s age, child’s gender, child’s chronic disease). The second part of the questionnaire included multiple-choice questions on the features of the paediatric emergency service and family physicians.

After the triage, the parents of all non-urgent patients (level-5) were invited to participate in the study. Questionnaires were handed out to the parents who agreed to participate in the study. Parents completed the questionnaires before the consultation at the PED. The data collection process took approximately 5–8 min for each person. One questionnaire was provided for each patient. During the study period, the parents who applied to the PED multiple times were not surveyed again.

### Ethics

Ethical approval was obtained from the Ethical Committee of Gaziantep University Medical Faculty in 03. 20. 2019 (protocol no.143). Informed consent was obtained from each participant.

### Data analysis

The SPSS 24.0 software package was used for statistical analyses and *p* < 0.05 was considered statistically significant. Descriptive statistics were expressed with numbers and percentages and chi-square test was used to compare analytical data.

## Results

### Population characteristics

A total of 479 patients and their parents were approached. Twenty-two parents declined to participate, leaving 457 for analysis. The average patient age was 6.5 ± 4.7 years and 24.5% had a chronic disease. Table 1 shows the demographic characteristics of the patients and their parents who were included in the study.

**Table 1.** Demographic characteristics of the patients and their parents.

Variable	N (%)
Age of the child	
Under 5	204 (44.6)
5–10	121 (26.5)
11–16	132 (28.9)
Gender of the child	
Male	197 (43.1)
Female	260 (56.9)
Age of the mother	
Under 35	199 (43.5)
35 and older	258 (56.5)
Age of the father	
Under 35	140 (30.6)
35 and older	317 (69.4)
Educational status of the mother	
Elementary School	123 (26.9)
Secondary School	127 (27.8)
High School	184 (40.3)
University	23 (5.0)
Educational status of the father	
Elementary School	154 (33.7)
Secondary School	125 (27.4)
High School	95 (20.8)
University	83 (18.2)
Level of Income of the family	
Low	119 (26)
Moderate	180 (39.4)
High	158 (34.6)

### Features of the non-urgent PED visits

Two hundred fifty-three (55.4%) of the patients presented outside working hours. The most important reasons for presenting to the PED are shown in Table 2.

**Table 2.** How parents use the paediatric emergency department.

	*N* (%)
Time of presentation to ED	
Within working hours	204 (44.6)
Outside working hours	253 (54.4)
Chronic disease status of the child	
Yes	112 (24.5)
No	345 (75.5)
The most important reason for taking children to the ED	
Upper respiratory tract problems	106 (23.2)
Gastrointestinal tract problems (abdominal pain-constipation-diarrhea-vomiting)	74 (16.2)
Complaints about worsened general condition (unease, crying, lack of appetite etc.)	73 (16)
Allergies	60 (13.1)
Fever	56 (12.3)
Urological complaints	27 (5.9)
Dermatological complaints	25 (5.5)
Muscle-bone pain	20 (4.4)
Other	16 (3.5)
Parents’ perception of emergency	
Very urgent	111 (24.3)
Moderately urgent	192 (42.0)
Less urgent	154 (33.7)
Duration of complaints	
Less than 1 h	48 (10.5)
1–24 h	242 (53)
Longer than 24 h	167 (36.5)
Did the parents take the child to the family physician before presenting to the emergency department?	
Yes	57 (12.5)
No	400 (87.5)
The number of visits to the emergency department within the last year	
Once	116 (27.5)
2–3 times	259 (56.7)
More than 3 times	82 (17.9)

One-third (33.7%) of the parents evaluated their children’s emergency status as very urgent. 53% of the complaints had been observed for 1–24 h. Of patients, 57 (12.5%) had been already seen by a family physician before their visit. Most of the children (74.6%) visited the PED more than once within the last year. The rate of children who did not visit their family physician within the last year was 10.3%. The most important reason for preferring PED (42.5%) instead of a family physician or alternative health facilities was the thought that the condition of children would ‘worsen’. Other reasons were as follows: 19.9% of the parents thought that the services provided in the ED were fast and reliable, 20.4% of the parents presented to the PED because their family physician and other physicians were not working at that time, and 17.3% thought that their child needed emergency care (Table 3).

**Table 3.** The reasons why non-urgent patients prefer the ED over alternative healthcare centres or family physician.

	n (%)
The thought that the complaints of their children would get worse	194 (42.5)
Family physician and other physicians not working at that time	93 (20.4)
The thought that emergency departments provide fast and reliable services	91 (19.9)
The thought that the child needs emergency healthcare	79 (17.3)

### Parents’ opinions on doctors

More than half (58.9%) of the parents who participated in the study stated that they were satisfied with their family physicians. On the other hand, only 32.2% (*N* = 147) of the parents indicated that they preferred their family physician, whereas most parents (67.8% *n* = 310) said that they preferred a paediatrician and other specialists when their children had a health problem.

### Explanatory analyses

We found a statistically significant association between the education level of fathers and a preference for other specialists rather than family physicians (*p* = 0.014). Elementary school graduates were more likely to prefer other specialists. There was no significant relationship between the level of satisfaction with the family physician and the frequency of using the PED (*p* > 0.05).

## Discussion

### Main findings

In this study, one-fifth of the children visiting the PED for non-urgent reasons (triage level 5), had a chronic disease. One-third of the parents (33.7%) presented to the ED because their family physician and other physicians were not working at that time. One**-**third of the parents evaluated the status of their child as ‘very urgent’. Nearly half of the parents (42.5%) thought that the complaints of their children would get worse. We showed that the majority of parents (68%) who use the PED for non-urgent patients prefer paediatricians and other specialists rather than their own family physicians for health problems in their children.

We found a statistically significant association between the education level of fathers and a preference for other specialists rather than family physicians. Fathers who had received only elementary education were more likely to prefer other specialists as compared to fathers who were better educated.

### Comparison with existing literature

Our study found that one-fifth of the children in the study had a chronic disease. The fact that the hospital at which our study was conducted is a tertiary care hospital that provides services for patients from nearby cities may have contributed to the high rate of patients with a chronic disease. Idil et al. [14] from Turkey reported that 25.5% of non-urgent adult patients had chronic disease. Similarly, Seo et al. [17] reported that 29.4% of the children presenting to ED had a chronic disease.

It was reported that factors such as age (≤5 years), male gender, foreign nationality, low socioeconomic status, cold weather, providing services outside working hours and factors related to access resulted in a higher number of patients presenting to EDs [18]. In this study, 44.6% of the non-urgent patients who presented to the PED were children under five years old. Most of the parents had an educational status lower than high school and an income level of low to moderate. In this study, it was also found that fathers with a low education level preferred other specialists instead of family physicians for their children’s health problems. Consistent with our findings, Kurt et al. [19] also reported that non-urgent admissions due to confidence in the ED were more frequent among parents who were elementary school graduate. The fact that the parents with a low educational status do not have sufficient knowledge on paediatric health may be the reason why they use ED for acute diseases. In a study Morrison et al. [20] reported that a low level of health literacy increased the number of non-urgent patients presenting to PEDs. Another study showed that non-urgent use of EDs could be significantly reduced by educational interventions provided for parents [21].

In this study, 58.9% of the parents stated that they were satisfied with their family physicians. On the other hand, it was found that only 32.2% of the parents preferred to visit their family physician when their child had a health problem. In one study, it was reported that Turkish families preferred paediatricians for routine follow-ups of their 5–15 years old children [22]. In a study reported that nearly one-third (28.7%) of the adult patients who presented to an ED with non-urgent complaints did not prefer their family physician for their health problems [14]. Another study found that 17.3% of non-urgent patients had no interaction with their family physicians [16]. Henninger et al. [23] reported that the main reason for the patients who first consult general practitioner was the quality of the relationship.

Our study also showed that the rate of children who were taken to their family physician before the ED was very low (12.5%). Smith et al. [24] reported that although most of the patients had a primary care physician, less than half of these patients contacted their primary care physician before presenting to the PED.

Parents’ concerns and perception of emergency and the clinicians’ evaluations differ in assessing illnesses in children. In our study, 24.3% of the parents considered their children’s emergency status less urgent, whereas most of the parents considered the same moderately urgent and very urgent. Kalidindi et al. [25] was found that although 94% of the parents who took their children to the ED considered their visit ‘urgent’, 27% of the physicians considered the same visits ‘non-urgent’.

It was found that the most important patient complaints were upper respiratory tract problems, gastrointestinal system problems and complaints about worsened general condition in our study. Similarly, in another study from Thailand reported that the patients presenting to the ED were most frequently diagnosed with acute respiratory tract infections, fever and gastroenteritis [26]. According to a study conducted in Belgium, the most important reasons for presenting to the ED were fever, respiratory tract problems and gastrointestinal problems [27].

In this study, the most important reason for preferring the PED was the parents’ thought that the complaints of their children would get worse. Other reasons for the preference of ED included accessibility outside working hours, quickness and reliability. Similar to our study, more than one-third of the parents (38.8%) stated that they went to the ED because they thought their child needed emergency care and the child’s condition was worsening in a study conducted in Lithuania [3]. In a study by Kubicek et al. [9], the most important causes for non-urgent patients presenting to the ED were found to be the belief that EDs provided better care for children, the trust in physicians and 24/7 access to EDs. According to another study from Singapore, the main reasons why caregivers brought non-urgent cases to the PED were the perception that the child’s symptoms would get worse, accessibility outside working hours, perceived advantages of the paediatricians at the hospital and lack of confidence in primary care physicians [12]. Results of our study are in keeping with the results of these studies.

### Strengths and limitations

To our knowledge, this is the first article to examine the characteristics of non-urgent PED visits and why family physicians are not used for these visits. Also, this study corroborates and extends findings from previous research on parents’ use of the ED for non-urgent paediatric healthcare needs. The limitation of our study is that it was conducted within a limited time (one month) in a single centre. Therefore, results of this study cannot be generalised. In addition, this study did not take into account seasonal variation in diseases.

## Conclusion

Parents’ perception of urgency and the thought that their child’s condition would worsen is an important reason for them to use the PED. Educating parents and improving the quality and accessibility of primary healthcare can reduce non-urgent PED visits.

## Ethics statement

Ethical approval was obtained from the Ethics Committee of Gaziantep University Medical Faculty.
